# Differential expression of chemokine receptors on peripheral blood B cells from patients with rheumatoid arthritis and systemic lupus erythematosus

**DOI:** 10.1186/ar1776

**Published:** 2005-06-22

**Authors:** Maren Henneken, Thomas Dörner, Gerd-Rüdiger Burmester, Claudia Berek

**Affiliations:** 1 Deutsches Rheuma ForschungsZentrum, Berlin, Germany; 2 Department of Rheumatology and Clinical Immunology, Charité, Humboldt University, Berlin, Germany

## Abstract

Chemokines and their receptors are essential in the recruitment and positioning of lymphocytes. To address the question of B cell migration into the inflamed synovial tissue of patients with rheumatoid arthritis (RA), peripheral blood naive B cells, memory B cells and plasma cells were analyzed for cell surface expression of the chemokine receptors CXCR3, CXCR4, CXCR5, CCR5, CCR6, CCR7 and CCR9. For comparison, B cells in the peripheral blood of patients with the autoimmune disease systemic lupus erythematosus (SLE) or with the degenerative disease osteoarthritis (OA) were analyzed. Expression levels of chemokine receptors were measured by flow cytometry and were compared between the different patient groups and healthy individuals. The analysis of chemokine receptor expression showed that the majority of peripheral blood B cells is positive for CXCR3, CXCR4, CXCR5, CCR6 and CCR7. Whereas a small fraction of B cells were positive for CCR5, practically no expression of CCR9 was found. In comparison with healthy individuals, in patients with RA a significant fraction of B cells showed a decreased expression of CXCR5 and CCR6 and increased levels of CXCR3. The downregulation of CXCR5 correlated with an upregulation of CXCR3. In patients with SLE, significant changes in CXCR5 expression were seen. The functionality of the chemokine receptors CXCR3 and CXCR4 was demonstrated by transmigration assays with the chemokines CXCL10 and CXCL12, respectively. Our results suggest that chronic inflammation leads to modulation of chemokine receptor expression on peripheral blood B cells. However, differences between patients with RA and patients with SLE point toward a disease-specific regulation of receptor expression. These differences may influence the migrational behavior of B cells.

## Introduction

Rheumatoid arthritis (RA) is a complex autoimmune disease of unknown etiology. It is characterized by chronic inflammation of the synovial membrane and the formation of a pannus, which leads to swollen joints and finally to joint destruction. Inflammatory cells such as monocytes and neutrophils, together with T and B cells, infiltrate the synovial membrane [1]. Migration of lymphocytes from the blood to the synovial tissue is a multi-step process controlled in part by interactions between chemokines and their receptors [2,3].

About 50 chemokines have been identified in humans and are divided into four groups according to their cysteine motifs [4]. After activation and differentiation, cells of the lymphoid lineages dynamically change their expression profiles of chemokine receptors, which results in specific migration in response to chemokines [5,6].

Under pathological conditions, such as RA, chemokines direct lymphocytes into the chronically inflamed synovial tissue [7,8]. Both the residual synovial-lining cells and infiltrating leukocytes are the source of pro-inflammatory chemokines such as CCL2, CCL3, CCL5, CCL20, CXCL9 and CXCL10, as well as chemokines important for the homeostasis of lymphocytes, such as CXCL12 and CXCL13 [2,9-13]. As a consequence of the synovial inflammation, these chemokines are also found in the synovial fluid.

Contradictory results have been reported for chemokine receptor expression on peripheral blood T cells of patients with RA [13,14] and even less is known about the expression of chemokine receptors on B cells. The classical chemokine receptor on B cells is CXCR5. Its ligand CXCL13 is a potent B cell chemo-attractant molecule directing B cells into the follicles of secondary lymphoid organs [15]. In addition to CXCR5, the chemokine receptors CXCR4 and CCR7 have been implicated in B cell migration into the follicular structures, and especially CXCR4 in directing plasma cells into the bone marrow [6]. These chemokines and their corresponding chemokine receptors are also expressed in the inflamed synovial tissue of patients with RA. In particular, high expression of CXCL13 was found when the synovial tissue contained large aggregates of B cells, resembling the structure of germinal centers [11,17]. These findings suggested that the chemokine CXCL13 is involved in B cell trafficking into the inflamed tissue and has a role in the formation of ectopic lymphoid tissue [18,19].

CXCR3 is a chemokine receptor for the inflammatory chemokines CXCL9, CXCL10 and CXCL11. It has been described as a marker for malignant B cells and is absent from the majority of normal peripheral blood B cells [20,21]. The analysis of patients with multiple sclerosis showed that CXCR3 becomes upregulated on infiltration of the inflamed cerebrospinal fluid [20].

To address the question of B cell migration, we analyzed chemokine receptor expression on peripheral blood B cells. Expression profiles of patients with RA were compared with those from patients with systemic lupus erythematosus (SLE), who – in contrast to patients with RA – usually show no or very little B cell accumulation at the site of chronic inflammation [22]. In addition, patients with osteoarthritis (OA) were included into the study as a control cohort. OA is a non-inflammatory degenerative joint disease. Our data suggest that the chronic inflammation in patients with RA and patients with SLE leads to changes in the chemokine receptor expression pattern on peripheral B cells; however, the expression patterns of chemokine receptors in RA and SLE are differentially regulated.

## Methods

### Patients and controls

Heparinized whole blood (9 ml) from patients with RA, patients with SLE and patients with OA was obtained from the Departments of Rheumatology and Orthopedics (Charité, Humboldt University, Berlin, Germany). Patients with RA were diagnosed in accordance with the American College of Rheumatology criteria [23]. As control, blood samples from healthy blood donors were analyzed. The demographic data and the treatment of patients are summarized in Table 1. The scientific ethics committee of Charité approved the study, and informed consent was obtained.

### Cell isolation and flow cytometry

Peripheral blood mononuclear cells (PBMC) were isolated by gradient centrifugation with Ficoll (Amersham Biosciences) and then stained (30 min at 4°C) with a biotin-coupled B cell-specific mAb against CD19 (clone SJ25-C1; Southern Biotechnology Associates), with the Cy5-labeled mAb against CD27 (clone 2E4; gift from RA van Lier, Academic Medical Center, Amsterdam) and with fluorescein isothiocyanate (FITC)-labeled mAb specific for one of the chemokine receptors CXCR3 (clone 49801.111; R&D Systems), CXCR5 (clone 51505.111; R&D Systems), CCR5 (clone 45523.111; R&D Systems), CCR6 (clone 53103.111; R&D Systems), CCR7 (clone 3D12; a gift from M Lipp, Max Delbrück Center, Berlin) or CCR9 (clone 112509.111; R&D Systems). Antibody against CXCR4 was labeled with phycoerythrin (PE; clone 12G5; BD Pharmingen). Before incubation with streptavidin-PE or streptavidin-FITC (for CXCR4) (0.5 μg/ml; Pharmingen), cells were washed twice in phosphate-buffered saline/2% BSA/4 mM EDTA.

To determine the frequency of CD5^+ ^B cells, PBMC were stained as described above with biotin-CD19, Cy5-CD27 and PE-labeled mAb against CD5 (clone UCHT2; BD Pharmingen). For the analysis of chemokine receptor expression, CD5^+ ^B cells were purified by magnetic cell sorting with CD19-specific beads (Miltenyi). Isolated B cells were stained as described above with PE-CD5 and Cy5-CD27 together with the FITC-labeled mAb against the chemokine receptors CXCR3 or CXCR5.

Propidium iodide (1 μg/ml; Sigma) was added immediately before cytometric analysis for the exclusion of dead cells. Flow cytometric analyses were performed by fluorescence-activated cell sorting (FACS) software (FACSCalibur and CellQuest; Becton Dickinson).

### Measurement of CXCL10 concentrations

For further analysis, sera of controls and those of patients analyzed for chemokine receptor expression were stored at -20°C. The concentration of CXCL10 was determined by using a sensitive ELISA test kit (HyCult Biotechnology). Samples were tested in duplicate and values were compared with a standard curve.

### Transmigration assay

Cell migration was examined in Transwell™ inserts (Corning Costar) with a diameter of 6.5 mm and 5 μm pores, by using fibronectin-precoated membranes as described previously [24]. In brief, 5 × 10^5 ^PBMC were suspended in RPMI 1640 medium (Life Technologies) without methyl red, supplemented with 0.5% BSA. Cells were added to the upper well and chemokine dilution or assay medium to the lower compartment; they were incubated for 90 min at 37°C under CO_2_-buffered conditions. Migrated cells from triplicate wells were pooled and analyzed by flow cytometry. Optimal chemokine concentrations for migration were 50 nM for CXCL12 (R&D Systems) and 100 nM for CXCL10 (R&D Systems).

### Statistical analyses

Statistical analyses were performed with GraphPad Prism software (Prism 3.0 software for windows; GraphPad). Frequencies of B cells were calculated with CellQuest software (Becton Dickinson) and variations in chemokine receptor expression on B cells within the analyzed group were compared in a hierarchic statistic analysis, using Kruskal–Wallis and the nonparametric Mann–Whitney *U *test. Correlations were determined by Spearman's product-moment correlation for interval data (GraphPad software). *P *< 0.05 was considered significant.

## Results

### Chemokine receptor expression on peripheral blood B cells of healthy controls

**Figure 1:**
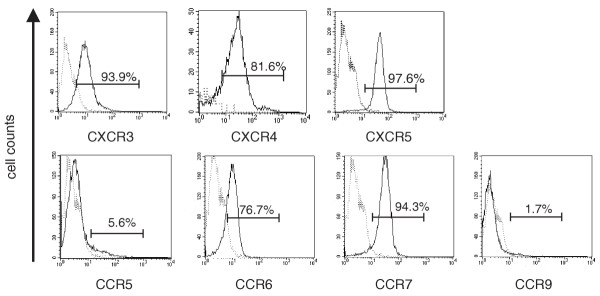
Expression of chemokine receptors in peripheral blood B cells from a healthy donor. Flow cytometric plots show chemokine receptor expression on CD19^+ ^B cells. For each of the chemokine receptors tested the percentage of positive B cells is indicated. Isotype control (dotted line) is included.

To analyze chemokine receptor expression on B cells of normal healthy volunteers PBMC were double-stained with antibodies specific for the B cell marker CD19 and for the chemokine receptors CXCR3, CXCR4, CXCR5, CCR5, CCR6, CCR7 or CCR9. Data from a representative experiment are depicted in Fig. 1. The cytometric analysis of healthy controls showed that 93.9% of the B cells were positive for CXCR3, 81.6% for CXCR4, 97.6% for CXCR5, 76.7% for CCR6 and 94.3% for CCR7. Most B cells were negative for the receptors CCR5 (only 5.6% positive) and CCR9 (only 1.7% positive).

### Analyses of chemokine receptor expression on peripheral blood B cell subsets of patients with RA, with SLE and with OA

**Figure 2:**
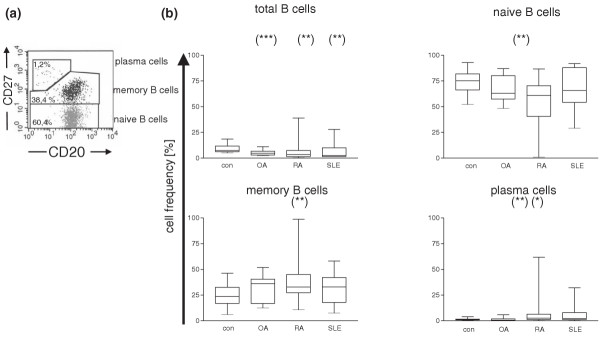
The frequency of B cells in healthy individuals and the different patient groups. **(a) **Triple staining with CD19, CD20 and CD27 was used to define naive B cells, memory B cells and plasma cells. Thresholds for CD27^-^, CD27^+ ^and CD27^hi ^are indicated. **(b) **Frequency for B cells and the different subpopulations. Data are given for healthy individuals (con) and for each of the patient groups analyzed: osteoarthritis (OA), rheumatoid arthritis (RA) and systemic lupus erythematosus (SLE). Box plots show the median values, 25th and 75th quartile and the range of values. Significant differences from controls are shown (*P < 0.05, **P < 0.005, ***P < 0.0005).

To assess changes in the pattern of B cell chemokine receptor expression associated with RA (*n *= 26), SLE (*n *= 11) and OA (*n *= 13) we compared the pattern in patients with that of a group of healthy individuals (*n *= 21). In addition to mAb specific for CD19 and the respective chemokine receptors, PBMC were stained with antibodies specific for CD27, which allowed us to distinguish between naive B cells (CD27^-^), memory B cells (CD27^+^) and plasma cells (CD27^hi^) [25]. Levels of CD27 expression used to define the various B cell subsets were defined by triple staining with CD19, CD20 and CD27 as shown in Fig. 2a. The relative frequencies of B cells are comparable in the control and in the different patient groups, although the patients with RA did show a higher variation in the percentages of naive B cells, memory B cells and plasma cells (Fig. 2b).

**Figure 3:**
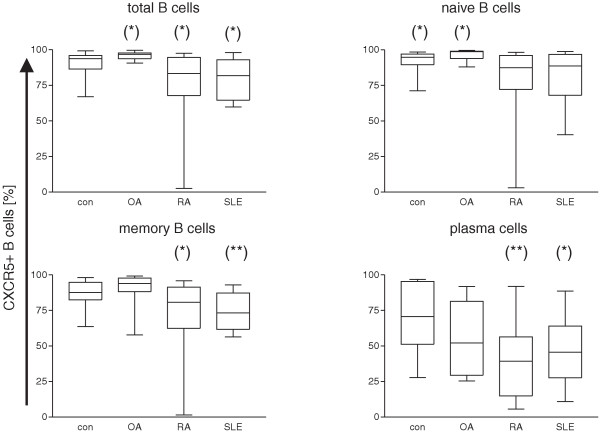
Chemokine receptor CXCR5^+ ^expression on B cell subpopulations. The percentage of total B cells, naive B cells, memory B cells and plasma cells expressing CXCR5^+ ^are given. Box plots show the median values, 25th and 75th quartile and the range of values. Significant differences from controls are shown (*P < 0.05, **P < 0.005).

In healthy individuals most B cells (median value 93.8%, range 66.9 to 99.3%) were positive for CXCR5 (CXCR5^+^; Fig. 3). Similar results were obtained with blood samples from patients with OA. As shown in Fig. 3 the percentage of CXCR5^+ ^B cells was slightly higher (median 96.8%, range 90.6 to 99.6%, *P *= 0.05). In both of these groups, only a minor fraction of B cells showed low expression of CXCR5. A representative FACS analysis showing CXCR5 expression on peripheral blood naive B cells, memory B cells and plasma cells from a healthy control is shown in Fig. 7.

In contrast to the patients with OA, a decrease in the fraction of CXCR5^+ ^B cells was observed in most blood samples from patients with RA and patients with SLE. In patients with RA the median value for CXCR5^+ ^B cells was decreased to 83.4% (*P *= 0.01) and in patients with SLE to 81.8% (*P *= 0.02) (Fig. 3). Whereas little variation in the frequency of CXCR5^+ ^B cells was seen in controls or in patients with OA, the expression of CXCR5 on B cells varied enormously in patients with RA and patients with SLE (Fig. 3). For patients with RA these values ranged from 2.5 to 97.6% and for patients with SLE from 59.8 to 98.1% (Fig. 3).

The analysis of the different B cell subsets revealed that the fraction of CXCR5^+ ^cells decreases as B cells differentiate to plasma cells. In healthy individuals the median value for the frequency of CXCR5^+ ^plasma cells was 70.6% (range 27.8 to 96.8%). In patients with RA and patients with SLE the expression of CXCR5 on peripheral blood plasma cells was reduced to 39.3% (range 5.7 to 91.9%) and 45.6% (range 11.0 to 88.6%), respectively (Fig. 3). Overall, in patients with RA significant differences in the fraction of CXCR5^+ ^B cells were found for naive B cells, memory B cells and plasma cells, whereas in patients with SLE this was seen only for effector B cells, memory B calls and plasma cells (Fig. 3).

**Figure 4:**
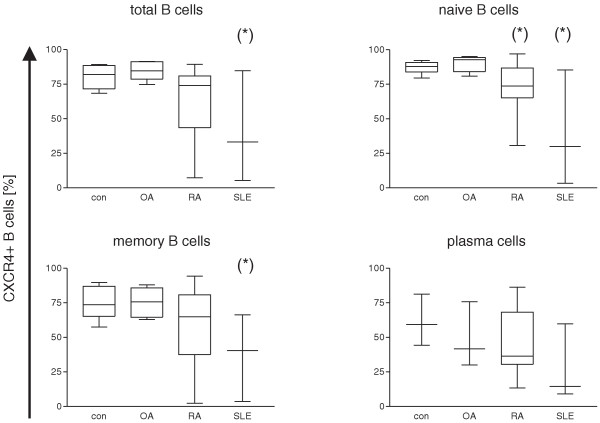
Chemokine receptor CXCR4^+ ^expression on B cell subpopulations. The percentage of total B cells, naive B cells, memory B cells and plasma cells expressing CXCR4^+ ^are given. Box plots show the median values, 25th and 75th quartile and the range of values. Significant differences from controls are shown (*P < 0.05).

Similar results were obtained when B cells were tested for the chemokine receptor CXCR4. In both healthy controls and patients with OA, most B cells expressed high levels of this chemokine receptor, and little individual variation was seen (Fig. 4). The median values for the fraction of CXCR4^+ ^B cells in healthy controls and patients with OA were 82.0% (range 68.4 to 89.3%) and 84.6% (range 74.7 to 91.5%), respectively. In contrast, in patients with RA (*n *= 19) the percentage of CXCR4^+ ^B cells was lower and large individual variations were found, from 7.3 to 89.4%. Similarly, in the few patients with SLE who were tested (*n *= 4) a reduction in the percentage of CXCR4^+ ^B cells was seen, ranging from 5.4 to 84.2% (Fig. 4).

**Figure 5:**
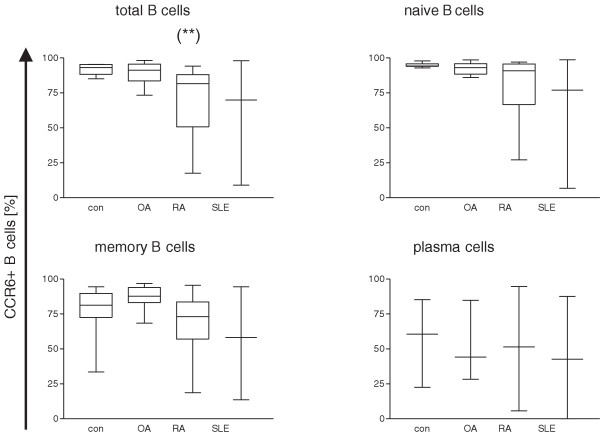
Chemokine receptor CCR6^+ ^expression on B cell subpopulations. The percentage of total B cells, naive B cells, memory B cells and plasma cells expressing CCR6^+ ^are given. Box plots show the median values, 25th and 75th quartile and the range of values. Significant differences from controls are shown (**P < 0.005).

When B cells from patients with RA and patients with SLE were tested for the expression of CCR6, again a reduction in the frequency of CCR6^+ ^B cells was observed. However, a significant reduction in the frequency of CCR6^+ ^B cells was found only when B cells were analyzed from patients with RA (*P *= 0.005; Fig. 5).

**Figure 6:**
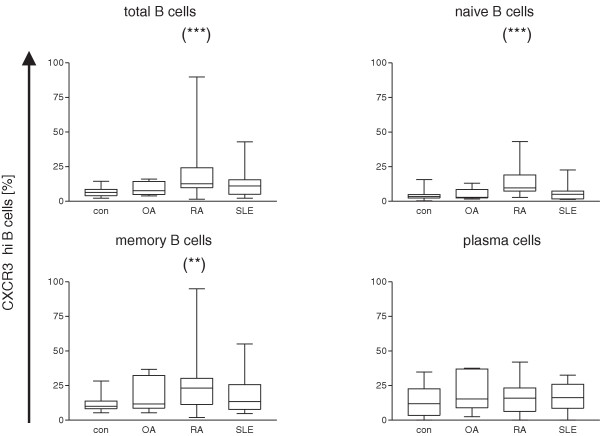
Chemokine receptor CXCR3^hi ^expression on B cell subpopulations. The percentage of total B cells, naive B cells, memory B cells and plasma cells expressing CXCR3^hi ^are given. Box plots show the median values, 25th and 75th quartile and the range of values. Significant differences from controls are shown (**P < 0.005, ***P < 0.0005).

**Figure 7:**
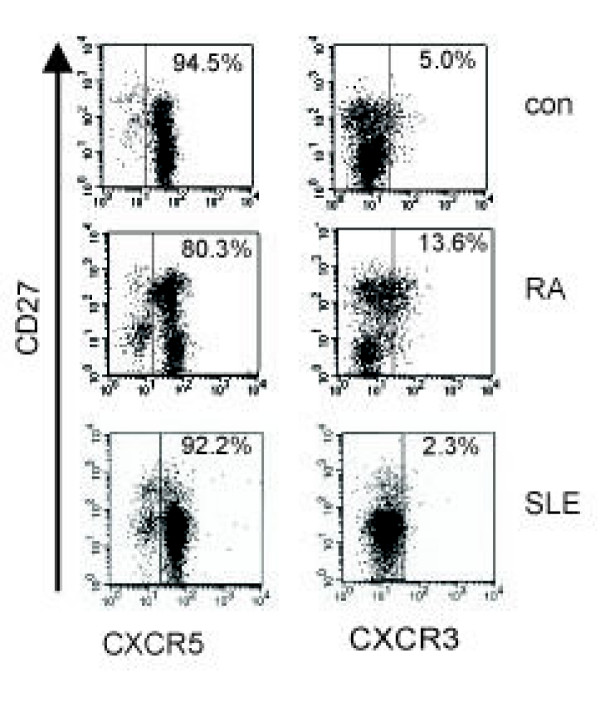
Modulation of CXCR3 and CXCR5 expression in patients. A representative fluorescence-activated cell sorting analysis for chemokine receptor expression on peripheral B cells (CD19^+^) is shown. The percentages of CXCR5^+ ^and CXCR3^hi ^B cells are given for healthy controls (con) and patients with osteoarthritis (OA), rheumatoid arthritis (RA) and systemic lupus erythematosus (SLE).

A great variation in chemokine receptor expression was obtained when B cells were tested for CXCR3, which is a receptor for chemokines that is upregulated in inflammation. We found that in healthy individuals most B cells are positive for CXCR3; however, the mean fluorescence intensity was rather low and only a few B cells expressed CXCR3, at a level comparable to that seen after staining with mAb specific for CXCR5 (Figs 1 and 7). The analysis of blood samples from patients with RA – and also from some patients with SLE – revealed an increase in the fraction CXCR3-high-expressing (CXCR3^hi^) B cells (Fig. 6). Again the percentage of CXCR3^hi ^B cells showed considerable variation between individual patients. In RA the values ranged from 1.6 to 89.8% (Fig. 6).

The results obtained for CXCR3 suggest that its expression is upregulated as B cells differentiate into memory B cells and plasma cells. In most healthy controls the fraction of CXCR3^hi ^naive B cells was negligible. A comparable result was found with blood samples from patients with OA and also with most patients with SLE. In contrast, in patients with RA a significant fraction of naive B cells showed high expression of CXCR3. The frequency was further increased in memory B cells from patients with RA (Figs 6 and 7). A comparison of B cells from healthy individuals and from patients with RA showed that the median value for naive B cells increased from 3.4% (range 0.4 to 15.7%) to 9.7% (range 2.8 to 43.1%) (*P *= 0.0006) and for memory cells from 10.0% (range 5.4 to 28.3%) to 23.2% (range 1.9 to 95.0%) (*P *= 0.003; Fig. 6). This highly significant increase in the fraction of CXCR3^hi ^B cells was not observed when blood samples from patients with SLE were analyzed.

**Figure 8:**
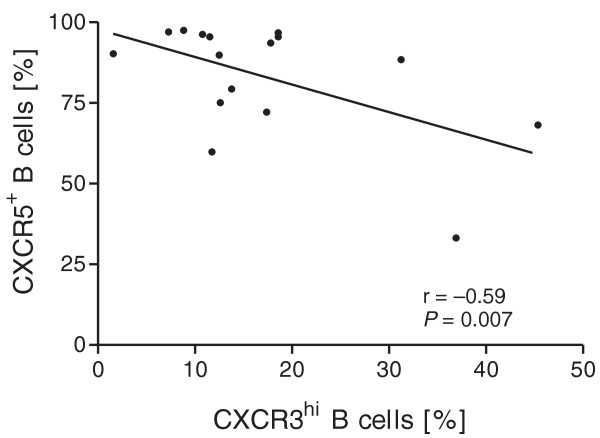
Negative correlation in the frequency of CXCR3^hi ^and CXCR5^+ ^B cells in patients with RA. Correlation factor (Spearman's) and *P *value are indicated (GraphPad software).

Furthermore, we addressed the question of whether there is a significant correlation between the expression of CXCR5 with that of CXCR4, CCR6 and CXCR3. In patients with RA a decreased fraction of CXCR5-expressing B cells correlated with the expression of CCR6 (*r *= 0.53, *P *= 0.01; data not shown). However, neither in patients with RA nor in patients with SLE did we see a significant correlation in the expression of CXCR5 and CXCR4. However, with regard to CXCR5 and CXCR3 we found a negative correlation, but this was restricted to patients with RA (*r *= -0.59, *P *= 0.007; Fig. 8).

### Chemokine receptor expression on CD5+ B cells

Staining of PBMC of healthy controls showed that on average 20% of B cells were positive for CD5 (data not shown). Comparable frequencies of CD5 expression were found for patients with RA (range 12 to 23% of B cells). To assess chemokine receptor expression on CD5^+ ^B cells, CD19^+ ^B cells were purified by magnetic cell sorting and stained for CD5, CD27 and the respective chemokine receptor. Labeling for CXCR5 showed that most CD5^+ ^B cells were positive for CXCR5 and only a fraction of CD5^+ ^plasma cells was negative for CXCR5 (data not shown). The frequency of CXCR3^hi ^B cells was the same for the CD5^+ ^and CD5^- ^B cell populations. There was therefore no evidence that CXCR3 expression is preferentially enhanced on CD5^+ ^B cells.

### The level of CXCL10 may influence CXCR3 expression

**Figure 9:**
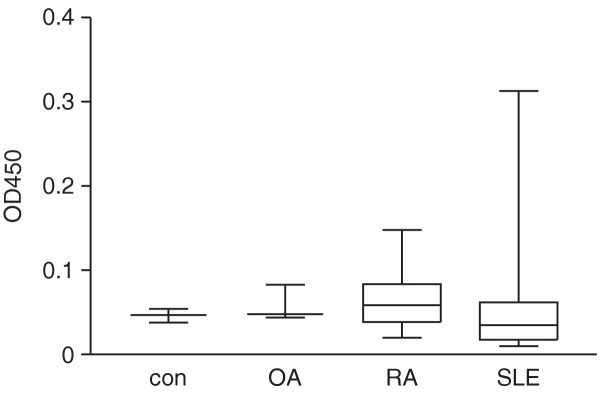
CXCL10 concentrations in sera. Sera of healthy controls and of patients with rheumatoid arthritis, systemic lupus erythematosus and osteoarthritis were tested in a standard ELISA test. A *D*_450 _of 0.06 corresponds to 200 pg/ml CXCL10 (concentration after Ficoll centrifugation); for each of the groups analyzed median values are indicated. Con, control; OA, osteoarthritis; RA, rheumatoid arthritis; SLE, systemic lupus erythematosus.

PBMC were isolated by Ficoll gradient centrifugation. In the supernatant the concentration of CXCL10 was measured with a standard ELISA test. In both sera from the different patient groups and from healthy controls only low levels of CXCL10 were detectable (about 200 pg/ml; Fig. 9). Only a single patient with SLE showed slightly increased levels of CXCL10.

### Influence of medication on chemokine receptor expression

**Figure 10:**
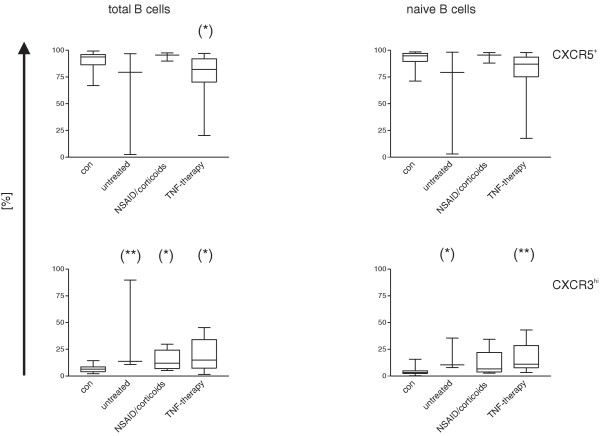
Effect of treatment on CXCR3 and CXCR5 expression. Data are shown for healthy individuals, untreated patients with rheumatoid arthritis and patients treated with corticoid and/or non-steroidal anti-inflammatory drugs or treatment with anti-tumor necrosis factor-α therapy. The percentage of CXCR3^hi ^and CXCR5^+ ^B cells is shown for total B cells and the subpopulation of naive B cells. Box plots show the median values, 25th and 75th quartile and the range of values. Significant differences from controls are shown (*P < 0.05, **P < 0.005). Con, control; RA, rheumatoid arthritis; SLE, systemic lupus erythematosus.

To determine a potential influence of medication, five blood samples of newly diagnosed patients with RA were analyzed before treatment. In three of the samples, B cells showed a pattern of chemokine receptor expression as described above, in that the median value for the frequency of B cells with high levels of CXCR5 was decreased from 93.8% (range 66.9 to 99.3%) to 79.4% (range 2.6 to 96.8%) (Fig. 10). Variations in chemokine receptor expression were also seen in patients (*n *= 10) under treatment with tumor necrosis factor (TNF) blockers (infliximab, etanercept and adalimumab). However, this was not found in patients with RA (*n *= 4) under treatment with corticoids and/or non-steroidal anti-inflammatory drugs (NSAID). In these patients practically all B cells were CXCR5^+ ^(median 95.5%, range 88.1 to 97.8%; Fig. 10).

The analysis of CXCR3 expression on B cells revealed that in comparison with healthy individuals, untreated patients with RA had an increase in the frequency of CXCR3^hi ^B cells. Most strikingly, an increase in the percentage of CXCR3^hi ^naive B cells, a finding characteristic for patients with RA, was observed. This increase was seen also under anti-TNF-α therapy and to some extent when patients where treated with corticoids and/or NSAID. The observed modulation of chemokine receptor expression on peripheral blood B cells from patients with RA therefore does not simply reflect the disease activity and/or the influence of therapy.

### The influence of chemokine receptor expression on B cell migration

**Figure 11:**
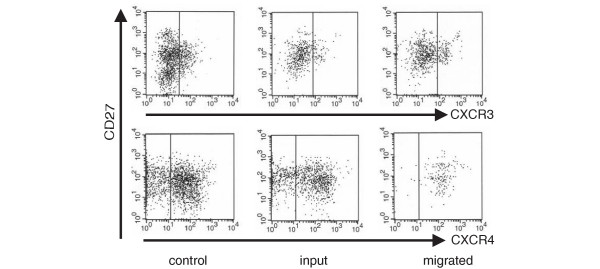
Chemokine receptor expression and B cell migration. Peripheral blood mononuclear cells were tested in a transmigration assay. A cytometric analysis showed CXCR3 (upper row) and CXCR4 (lower row) expression on CD19^+ ^B cells before analysis (control), after 90 min incubation in medium without chemokines (input) and after migration towards the chemokines CXCL10 (upper row) or CXCL12 (lower row) respectively (migrated).

To test whether low expression of CXCR3 on B cells is sufficient to respond to the inflammatory chemokine CXCL10, PBMC from patients with RA were tested with the use of a transmigration assay (Fig. 11). A cytometric analysis of the migrated cells revealed that both fractions of memory B cells, CXCR3^hi ^and CXCR3^lo ^B cells, migrated toward CXCL10 (Fig. 11). In contrast, in an analysis of migration towards the chemokine CXCL12, only CXCR4^+ ^B cells responded; CXCR4^- ^B cells did not (Fig. 11). The *in vitro *chemotaxis assay suggested that CXCR3-low-expressing B cells are functionally reactive towards the chemokine CXCL10.

## Discussion

The analysis of peripheral blood B cells from patients with RA and patients with SLE showed significant differences in their chemokine receptor expression when compared with B cells from healthy individuals and also from patients with OA. In patients with RA and patients with SLE a fraction of B cells showed decreased expression of CXCR5, CXCR4 and CCR6, chemokine receptors that have been associated with B cell homing into follicles [26,27]. In contrast, the expression of CXCR3, a receptor reactive to inflammatory chemokines [4,10], was increased. These changes in chemokine receptor expression seem to be associated with chronic inflammation, because they were not observed when B cells from patients with OA were analyzed. Importantly, a comparison of B cells from patients with RA and patients with SLE showed distinct disease signatures. A negative correlation of CXCR5 and CXCR3 expression in B cells was seen only in patients with RA.

CXCR5 was previously shown to be expressed on most mature circulating B cells [8]. This receptor is the main chemokine receptor responsible for the controlled migration of B cells into secondary lymphoid organs [28]. The analysis of blood samples from healthy individuals showed that after activation of B cells and their differentiation into plasma cells and to some extent into memory cells, CXCR5 becomes downregulated (Fig. 3). In line with these results are data from an experiment *in vitro *in which stimulation with anti-CD40 antibodies led to a downregulation of CXCR5 [29]. The finding of a significant decrease in the fraction of CXCR5^+ ^B cells in patients with RA and also in patients with SLE by our study may reflect the chronic activation of B cells as reported for these groups of patients [30].

Lower levels of chemokine receptor CXCR5 and higher levels of CXCR3 on peripheral blood B cells may represent a generalized change in the profile and might be seen on all of the activated B cells in the systemic compartment or these changes in chemokine receptor expression might serve in selective recruitment into the inflamed tissue. In line with this interpretation we observed CXCR3 expression on synovial tissue B cells. However, comprehensive studies are under way to further delineate the expression of chemokine receptors and their function in migration into the effected tissue.

CXCR3 was described as a marker for malignant B cells, and its expression on normal peripheral blood B cells has been controversial [20,21]. Our results show that practically all B cells express CXCR3, although with a low mean fluorescence intensity. After differentiation, the level of CXCR3 expression seems to be upregulated because the frequency of CXCR3^hi ^B cells increases from naive to memory B cells. Activation of human B cells using a culture system *in vitro *showed that after stimulation with cytokines the expression of CXCR3 is upregulated [31]. Similarly, it was shown for T lymphocytes that after activation with interleukin-2 the expression of CXCR3 is upregulated [32]. The observed increase in the frequency of CXCR3^hi ^B cells may result from the chronic activation and differentiation of B cells in patients with RA.

A significant increase in the fraction of CXCR3^hi ^B cells was observed only when blood samples from patients with RA were analyzed (Fig. 6). In sera of patients with SLE but not in those from patients with RA, Narumi and colleagues [33] described high titers of CXCL10. Because CXCR3 expression may be influenced by the level of CXCL10, sera from healthy controls and from the different patient groups were tested for the presence of this chemokine. However, we did not find elevated titers of CXCL10 in our patients with SLE (Fig. 9). These results exclude the possibility that a ligand-induced receptor internalization might underlie the lower frequency of CXCR3^hi^-expressing B cells in patients with SLE.

Using a transmigration assay we were able to show that low levels of CXCR3 expression are sufficient to permit a response to the migrational stimulus of the chemokine CXCL10. However, these chemotaxis results *in vitro *are not necessarily predictive of their lymphocyte-recruiting activity *in vivo*. The different levels of chemokine receptor CXCR3 on peripheral blood B cells may still affect B cell migration. Elevated levels of the interferon-γ-inducible chemokines CXCL9 and CXCL10, both ligands for CXCR3, have been found in chronically inflamed synovial tissue [10]. These chemokines, which are normally involved in the chemotaxis of neutrophils, T cells and mast cells, might also influence the migration of CXCR3^+ ^B cells. Further experiments will be required to show whether the significant upregulation of CXCR3 on peripheral blood B cells supports their accumulation in the inflamed synovial tissue.

Whereas B cells from healthy controls and patients with OA showed little inter-individual variation in the expression of chemokine receptors, individual patients with RA and patients with SLE gave a rather heterogeneous picture (Fig. 7). For each of the chemokine receptors analyzed, the fractions of negative, low and highly positive B cells varied tremendously and were seen on both B cells and memory cells.

Little is known about the mechanisms controlling chemokine receptor expression and what might cause the variability in their expression level on peripheral blood B cells. One possibility might be that the modulation of chemokine receptor expression is associated with rheumatoid factor (RF) antibody titers. The majority of patients with RA analyzed were positive for RF. A correlation of chemokine receptor expression and the level of RF was therefore not seen.

An attempt to correlate the level of chemokine receptor expression with age or sex of the patients, with disease duration or with disease activity failed. There was no clear-cut correlation to be seen. Furthermore, from our data it seems unlikely that the variability in chemokine receptor expression results from the different treatment regimes of individual patients with RA (Fig. 10). Individual variability of chemokine receptor expression on B cells was as great in recently diagnosed, yet untreated, patients with RA as in those receiving anti-TNF-α therapies, which suggests that TNF-α itself is unlikely to be the cause of receptor modulation.

RA and SLE are chronic inflammatory diseases, and both are characterized by a continuous activation of B cells. Whereas SLE is a more systemic inflammatory disease, in most patients with RA the inflammation is localized primarily to the synovial membrane. To what extent the described differences in chemokine receptor expression between B cells from patients with RA and patients with SLE might influence the migrational pattern of B cells needs to be delineated by continuing studies, potentially permitting new therapeutic avenues.

## Conclusion

Here we show that chronic inflammation influences chemokine receptor expression on peripheral blood B cells. Receptors for homeostatic chemokines, like CXCL13 and CXCL12 showed reduced levels of expression whereas CXCR3 a receptor for inflammatory chemokines becomes unregulated. Differences between RA and SLE patients suggest a disease specific regulation of chemokine receptor expression, which may influence the migrational behavior of B cells.

## Abbreviations

BSA = bovine serum albumin; ELISA = enzyme-linked immunosorbent assay; FITC = fluorescein isothiocyanate; FACS = fluorescence-activated cell sorting, mAb = monoclonal antibody; OA = osteoarthritis; PBMC = peripheral blood mononuclear cells; PE = phycoerythrin; RA = rheumatoid arthritis; RF = rheumatoid factor; SLE = systemic lupus erythematosus; TNF = tumor necrosis factor.

## Competing interests

The author(s) declare that they have no competing interests.

## Authors' contributions

MH made acquisition of data and their interpretation, performed the statistical analysis and drafted the article. TD was responsible for assessment of patients and revising the article critically. G-RB was involved in the analysis and careful discussion of the data. CB coordinated the study, was involved in the critical discussion of results and their interpretation and helped to draft the article. All authors read and approved the final manuscript.

## Figures and Tables

**Table 1:** Demographics and medications of patients in the study

Parameter	RA	SLE	OA	Controls
Total numbers	26	11	13	21
Age (years)				
Mean	55.1	42.5	71.2	42.6
Range	25–76	27–78	62–79	25–64
Sex (*n*)				
Female	21	10	8	15
Male	5	1	5	6
Duration of disease (years)				
Mean	8.1	9.5	3.3	-
Range	0.1–37	2–25	0.5–10	-
Clinical parameter (mean ± SD)				
Leukocytes (10^9^/l)	9.5 ± 2.7	4.3 ± 1.1	7.8 ± 2.7	nd
CRP (mg/dl)	3.1 ± 3.0	1.1 ± 0.9	0.5 ± 0.6	nd
ESR (mm/h)	37.5 ± 30.5	34.9 ± 16.7	28.5 ± 19.4	nd
Treatment (*n*)				
None	6	-	-	21
Corticosteroids and/or NSAID	7	-	8^a^	-
Anti-TNF-α therapy	13^b^	-	-	-
Predisolone	-	11^c^	-	-
Cyclophosphamide (i.v.)	-	6	-	-

CRP, C-reactive protein; ESR, erythrocyte sedimentation rate; i.v., intravenous; nd, not determined; NSAID, non-steroidal anti-inflammatory drugs; OA, osteoarthritis; RA, rheumatoid arthritis; SLE, systemic lupus erythematosus.

^a^Treatment with buprenorphine (1) or tramadol (1); ^b^treatment with TNF-α-specific antibodies infliximab (6) or adalimumab (3) (mostly in combination with methotrexate) or with the soluble TNF-α receptor etanercept (2); ^c^treatment with predisolone alone (2), in combination with azathioprine (3), or in combination with cyclophosphamide (6).
